# Language and communication in international students’ adaptation: a bibliometric and content analysis review

**DOI:** 10.1007/s10734-022-00888-8

**Published:** 2022-07-12

**Authors:** Michał Wilczewski, Ilan Alon

**Affiliations:** 1 Faculty of Applied Linguistics, University of Warsaw, Dobra 55, 00-312 Warsaw, Poland; 2 Department of Economics and Business Administration, University of Ariel, 40700 Ariel, Israel; 3 School of Business and Law, University of Agder, Gimlemoen 25, 4630 Kristiansand, Norway

**Keywords:** Literature review, Language, Intercultural communication, International student, Adaptation, Bibliometric analysis

## Abstract

This article systematically reviews the literature (313 articles) on language and communication in international students’ cross-cultural adaptation in institutions of higher education for 1994–2021. We used bibliometric analysis to identify the most impactful journals and articles, and the intellectual structure of the field. We used content analysis to synthesize the results within each research stream and suggest future research directions. We established two major research streams: second-language proficiency and interactions in the host country. We found inconclusive results about the role of communication with co-nationals in students’ adaptation, which contradicts the major adaptation theories. New contextualized research and the use of other theories could help explain the contradictory results and develop the existing theories. Our review suggests the need to theoretically refine the interrelationships between the interactional variables and different adaptation domains. Moreover, to create a better fit between the empirical data and the adaptation models, research should test the mediating effects of second-language proficiency and the willingness to communicate with locals. Finally, research should focus on students in non-Anglophone countries and explore the effects of remote communication in online learning on students’ adaptation. We document the intellectual structure of the research on the role of language and communication in international students’ adaptation and suggest a future research agenda.

## Introduction

One of the consequences of globalization is the changing landscape of international higher education. Over the past two decades, there has been a major increase in the number of international students, that is, those who have crossed borders for the purpose of study (OECD, 2021a), from 1.9 million in 1997 to over 6.1 million in 2019 (UIS Statistics, 2021). Even students who are motivated to develop intercultural competence by studying abroad (Jackson, 2015) face several challenges that prevent them from benefitting fully from that experience. Examples of these challenges include language and communication difficulties, cultural and educational obstacles affecting their adaptation, socialization, and learning experiences (Andrade, 2006), psychological distress (Smith & Khawaja, 2011), or social isolation and immigration and visa extension issues caused by Covid-19 travel restrictions (Hope, 2020).

**Fig. 1:**
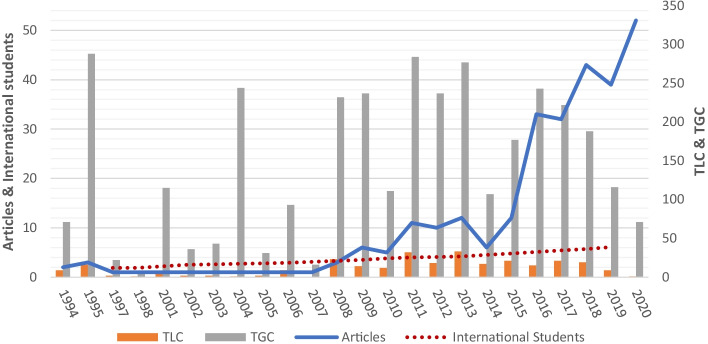
Yearly publication of articles on language and communication in student adaptation ( Source: HistCite). *Note*. TLC, total local citations received; TGC, total global citations received; Articles, number of articles published in the field; International Students, number (in millions) of international students worldwide (UIS Statistics, 2021)

Cross-cultural adaptation theories and empirical research (for reviews, see Andrade, 2006; Smith & Khawaja, 2011) confirm the critical importance of foreign-language and communication skills and transitioning to the host culture for a successful academic and social life. Improving our understanding of the role of foreign-language proficiency and communication in students’ adaptation is important as the number of international students in higher education worldwide is on the rise. This increase has been accompanied by a growing number of publications on this topic over the last decade (see Fig. 1). Previous reviews of the literature have identified foreign-language proficiency and communication as predictors of students’ adaptation and well-being in various countries (Smith & Khawaja, 2011). The most recent reviews (Jing et al., 2020) list second-language acquisition and cross-cultural adaptation as among the most commonly studied topics in international student research. However, to date, there are no studies specifically examining the role of *language and communication in international students’ adaptation* (henceforth “language and communication in student adaptation”). This gap is especially important given recent research promoting students’ self-formation (Marginson, 2014) and reciprocity between international and domestic students (Volet & Jones, 2012). The results challenge the traditional “adjustment to the host culture” paradigm whereby international students are treated as being out of sync with the host country’s norms (Marginson, 2014). Thus, this article differs from prior research by offering a systematic and in-depth review of the literature on language and communication in student adaptation using bibliometric co-citation analysis and qualitative content analysis. Our research has a methodological advantage in using various bibliometric tools, which should improve the validity of the results.

We focus on several questions:

1. What are the most impactful journals and articles about the role of language and communication in student adaptation?
2. What is the thematic structure of the research in the field?
3. What are the leading research streams investigating language and communication in student adaptation?
4. What are the effects of language and communication on student adaptation?
5. What are the future research directions?

After introducing the major concepts related to language and communication in student adaptation and the theoretical underpinnings of the field, we present our methodology. Using bibliometric and content analysis, we track the development of the field and identify the major themes, research streams, and studies that have shaped the state-of-the art and our current knowledge about the role of language and communication in student adaptation. Finally, we suggest avenues for future research.

## Defining the concepts and theories related to language and communication in student adaptation

### Concepts related to language and communication

Culture is a socially constructed reality in which language and social practices interact to construct meanings (Burr, 2006). In this social constructionist perspective, *language* is viewed as a form of social action. Intertwined with culture, it allows individuals to communicate their knowledge about the world, as well as the assumptions, opinions, and viewpoints they share with other people (Kramsch, 1998). In this sense, people identify themselves and others through the use of language, which allows them to communicate their social and cultural identity (Kramsch, 1998).

*Intercultural communication* refers to the process of constructing shared meaning among individuals with diverse cultural backgrounds (Piller, 2007). Based on the research traditions in the language and communication in student adaptation research, we view *foreign or second-language proficiency*, that is, the skill allowing an individual to manage communication interactions in a second language successfully (Gallagher, 2013), as complementary to communication (Benzie, 2010).

### Cross-cultural adaptation

The term *adaptation* is used in the literature interchangeably with *acculturation*, *adjustment*, *assimilation*, or *integration*. Understood as a state, *cultural adaptation* refers to the degree to which people fit into a new cultural environment (Gudykunst and Hammer, 1988), which is reflected in their psychological and emotional response to that environment (Black, 1990). In processual terms, adaptation is the process of responding to the new environment and developing the ability to function in it (Kim, 2001).

The literature on language and communication in student adaptation distinguishes between psychological, sociocultural, and academic adaptation. *Psychological adaptation* refers to people’s psychological well-being, reflected in their satisfaction with relationships with host nationals and their functioning in the new environment. *Sociocultural adaptation* is the individual’s ability to fit into the interactive aspects of the new cultural environment (Searle and Ward, 1990). Finally, *academic adaptation* refers to the ability to function in the new academic environment (Anderson, 1994). We will discuss the results of the research on language and communication in student adaptation with reference to these adaptation domains.

### Theoretical underpinnings of language and communication in student adaptation

We will outline the major theories used in the research on international students and other sojourners, which has recognized foreign-language skills and interactions in the host country as critical for an individual’s adaptation and successful international experience.

The sojourner adjustment framework (Church, 1982) states that host-language proficiency allows one to establish and maintain interactions with host nationals, which contributes to one’s adaptation to the host country. In turn, social connectedness with host nationals protects one from psychological distress and facilitates cultural learning.

The cultural learning approach to acculturation (Ward et al., 2001) states that learning culture-specific skills allows people to handle sociocultural problems. The theory identifies foreign-language proficiency (including nonverbal communication), communication competence, and awareness of cultural differences as prerequisites for successful intercultural interactions and sociocultural adaptation (Ward et al., 2001). According to this approach, greater intercultural contact results in fewer sociocultural difficulties (Ward and Kennedy, 1993).

Acculturation theory (Berry, 1997, 2005; Ward et al., 2001) identifies four acculturation practices when interacting with host nationals: assimilation (seeking interactions with hosts and not maintaining one’s cultural identity), integration (maintaining one’s home culture and seeking interactions with hosts), separation (maintaining one’s home culture and avoiding interactions with hosts), and marginalization (showing little interest in both maintaining one’s culture and interactions with others) (Berry, 1997). Acculturation theory postulates that host-language skills help establish supportive social and interpersonal relationships with host nationals and, thus, improve intercultural communication and sociocultural adjustment (Ward and Kennedy, 1993).

The anxiety/uncertainty management (AUM) theory (Gudykunst, 2005; Gudykunst and Hammer, 1988) states that intercultural adjustment is a function of one’s ability to cope with anxiety and uncertainty caused by interactions with hosts and situational processes. People’s ability to communicate effectively depends on their cognitive resources (e.g., cultural knowledge), which helps them respond to environmental demands and ease their anxiety.

The integrative theory of communication and cross-cultural adaptation (Kim, 2001) posits that people’s cultural adaptation is reflected in their functional fitness, meaning, the degree to which they have internalized the host culture’s meanings and communication symbols, their psychological well-being, and the development of a cultural identity (Kim, 2001). Communication with host nationals improves cultural adaptation by providing opportunities to learn about the host country’s society and culture, and developing intercultural communication competence that includes the ability to receive and interpret comprehensible messages in the host environment.

The intergroup contact theory (Allport, 1954; Pettigrew, 2008) states that contact between two distinct groups reduces mutual prejudice under certain conditions: when groups have common goals and equal status in the social interaction, exhibit intergroup cooperation, and have opportunities to become friends. Intercultural contact reduces prejudice toward and stereotypical views of the cultural other and provides opportunities for cultural learning (Allport, 1954).

These theories provide the theoretical framework guiding the discussion of the results synthesized through the content analysis of the most impactful articles in the field.

## Methodology

### Bibliometric and content analysis methods

We used a mixed-method approach to review the research on language and communication in student adaptation for all of 1994–2021. This timeframe was informed by the data extraction process described in the next section. Specifically, we conducted quantitative bibliometric analyses such as co-citation analysis, keyword co-occurrence analysis, and conceptual thematic mapping, as well as qualitative content analysis to explore the research questions (Bretas & Alon, 2021).

Bibliometric methods use bibliographic data to identify the structures of scientific fields (Zupic and Čater, 2015). Using these methods, we can create an objective view of the literature by making the search and review process transparent and reproducible (Bretas and Alon, 2021). First, we measured the impact of the journals and articles by retrieving data from HistCite concerning the number of articles per journal and citations per article. We analyzed the number of total local citations (TLC) per year, that is, the number of times an article has been cited by other articles in the same literature (313 articles in our sample). We then analyzed the total global citations (TGC) each article received in the entire Web of Science (WoS) database. We also identified the trending articles in HistCite by calculating the total citation score (TLCe) at the end of the year covered in the study (mid-2021). This score rewards articles that received more citations within the last three years (i.e., up to the beginning of 2018). Using this technique, we can determine the emerging topics in the field because it considers not only articles with the highest number of citations received over a fixed period of time, but also those that have been cited most frequently in recent times (Alon et al., 2018).

Second, to establish a general conceptual structure of the field, we analyzed the co-occurrence of authors’ keywords using VOS software. Next, based on the authors’ keywords, we plotted a conceptual map using Biblioshiny (a tool for scientific mapping analysis that is part of the R bibliometrix-package) to identify motor, basic, niche, and emerging/declining themes in the field (Bretas and Alon, 2021).

Third, to determine specific research streams and map patterns within the field (Alon et al., 2018), we used the co-citation mapping techniques in HistCite that analyze and visualize citation linkages between articles (Garfield et al., 2006) over time.

Next, we used content analysis to synthesize the results from the 31 most impactful articles in the field. We analyzed the results within each research stream and discussed them in light of the major adaptation theories to suggest future research directions and trends within each research stream (Alon et al., 2018). Content analysis allows the researcher to identify the relatively objective characteristics of messages (Neuendorf, 2002). Thus, this technique enabled us to verify and refine the results produced by the bibliometric analysis, with the goal of improving their validity.

### Data extraction

We extracted the bibliographic data from Clarivate Analytics’ WoS database that includes over 21,000 high-quality, peer-reviewed scholarly journals (as of July 2020 from clarivate.libguides.com). We adopted a two-stage data extraction approach (Alon et al., 2018; Bretas and Alon, 2021). Table 1 describes the data search and extraction processes.

**Table 1:** Keyword search in WoS

Step	Keyword search	# Articles
1	(("international student*" OR "foreign student*" OR "overseas student*" OR “study* abroad” OR “international education”) AND (("language*" OR "communicat*") AND ("adapt*" OR "adjust*" OR "integrat*" OR “acculturat*”))) **Refined by: LANGUAGES: (ENGLISH) AND DOCUMENT TYPES: (ARTICLE)**	520
2	((("international student*" OR "foreign student*" OR "overseas student*" OR "study* abroad" OR "international education") AND ("language*" OR "communicat*") AND ("adapt*" OR "adjust*" OR "integrat*" OR "acculturat*" OR "identity" OR "satisf*"))) **Refined by:** **LANGUAGES:** (ENGLISH) AND **DOCUMENT TYPES:** (ARTICLE)	901
3	(((("international student*" OR "foreign student*" OR "overseas student*" OR "study* abroad" OR "international education") AND ("language*" OR "communicat*") AND ("adapt*" OR "adjust*" OR "integrat*" OR "acculturat*" OR "identit*" OR "satisf*" OR "cultur* shock")))) **Refined by:** **LANGUAGES:** (ENGLISH) AND **DOCUMENT TYPES:** (ARTICLE)	921
4	***The initial analysis of 921 articles resulted in removing studies other than research articles, those that focused on the experiences of students outside the host country or the experiences of other stakeholders, used the terms “adaptation”, “adjustment”, “integration”, or “identity” in senses other than those related to adaptation theory, and did not focus on language and communication as independent variables in their adaptation models***	313

First, in June 2021, we used keywords that would best cover the researched topic by searching for the following combinations of terms: (a) “international student*” OR “foreign student*” OR “overseas student*” OR “study* abroad” OR “international education”—to cover international students as a specific sojourner group; (b) “language*” and “communicat*”—to cover research on foreign-language proficiency as well as communication issues; and (c) “adapt*” OR “adjust*” OR “integrat*” OR “acculturat*”—to cover the adaptation aspects of the international students’ experience. However, given that cross-cultural adaptation is reflected in an individual’s functional fitness, psychological well-being, and development of a cultural identity (Kim, 2001), we included two additional terms in the search: “identit*” OR “satisf*”—to cover the literature on the students’ identity issues and satisfaction in the host country. Finally, based on a frequency analysis of our data extracted in step 2, we added “cultur* shock” in step 3 to cover important studies on culture shock as one of critical aspects of cross-cultural adaptation (Gudykunst, 2005; Pettigrew, 2008; Ward et al., 2001). After refining the search by limiting the data to articles published in English, the extraction process yielded 921 sources in WoS.

In the second stage, we refined the extraction further through a detailed examination of all 921 sources. We carefully read the articles’ abstracts to identify those suitable for further analysis. If the abstracts did not contain one or more of the three major aspects specified in the keyword search (i.e., international student, language and communication, adaptation), we studied the whole article to either include or exclude it. We did not identify any duplicates, but we removed book chapters and reviews of prior literature that were not filtered out by the search in WoS. Moreover, we excluded articles that (a) reported on students’ experiences outside of higher education contexts; (b) dealt with teaching portfolios, authors’ reflective inquiries, or anecdotal studies lacking a method section; (c) focused on the students’ experience outside the host country or on the experience of other stakeholders (e.g., students’ spouses, expatriate academics); (d) used the terms “adaptation,” “integration,” or “identity” in a sense different from cultural adaptation (e.g., adaptation of a syllabus/method/language instruction; integration of research/teaching methods/technology; “professional” but not “cultural” identity); or (e) used language/communication as a dependent rather than an independent variable. This process yielded 313 articles relevant to the topic. From them, we extracted the article’s title, author(s) names and affiliations, journal name, number, volume, page range, date of publication, abstract, and cited references for bibliometric analysis.

In a bibliometric analysis, the article is the unit of analysis. The goal of the analysis is to demonstrate interconnections among articles and research areas by measuring how many times the article is (co)cited by other articles (Bretas & Alon, 2021).

## Bibliometric analysis

### Most relevant journals and articles

**Fig. 2:**
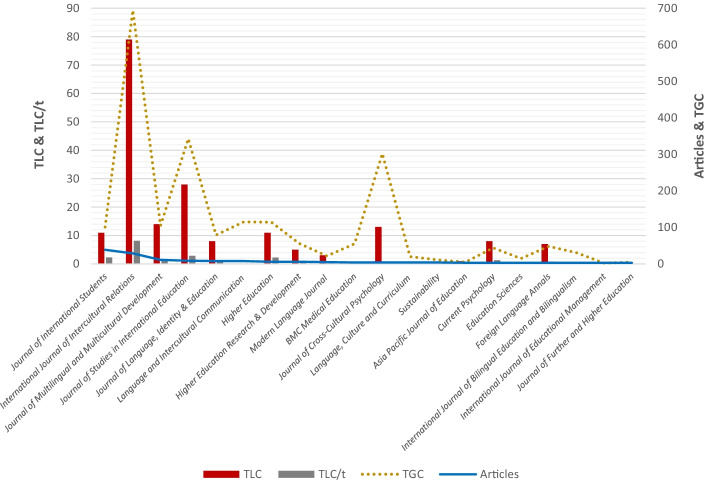
Top 20 journals publishing on language and communication in student adaptation ( Source: HistCite). *Note*. TLC, total local citations received; TLC/t, total local citations received per year; TGC, total global citations received; Articles, number of articles published in the field

We addressed research question 1 regarding the most impactful journals and articles about the role of language and communication in student adaptation by identifying the most relevant journals and articles. Figure 2 lists the top 20 journals publishing in the field. The five most influential journals in terms of the number of local and global citations are as follows: *International Journal of Intercultural Relations* (79 and 695 citations, respectively), *Journal of Studies in International Education* (28 and 343 citations, respectively), *Journal of Multilingual and Multicultural Development* (14 and 105 citations, respectively), *Journal of Cross-Cultural Psychology* (13 and 302 citations, respectively), and *Higher Education* (11 and 114 citations, respectively),

Table 2 lists the 20 most influential and trending articles as measured by, respectively, local citations (TLC) and trending local citations at the end of the period covered (TLCe), that is, mid-2021. The most locally cited article was a qualitative study of Asian students’ experiences in New Zealand by Campbell and Li (2008) (TLC = 12). That study, which linked host-language proficiency with student satisfaction and effective communication in academic contexts, also received the highest number of global citations per year (TGC/t = 7.86). The most influential article in terms of total local citations per year was a quantitative study by Akhtar and Kröner-Herwig (2015) (TLC/t = 1.00) who linked students’ host-language proficiency, prior international experience, and age with acculturative stress among students in Germany. Finally, Sam’s (2001) quantitative study, which found no relationship between host-language and English proficiency and having a local friend on students’ satisfaction with life in Norway, received the most global citations (TGC = 115).

**Table 2:** Ranking of the 20 most impactful and trending articles (sorted by TLC)

Rank	Author(s)/year	TLC	TLC/t	TGC	TGC/t	TLCe
1	Campbell and Li (2008)	12	0.86	110	7.86	4
2	Swami et al. (2010)	10	0.83	21	1.75	3
3	Duru and Poyrazli (2011)	10	0.91	28	2.55	7
4	Yang et al. (2006)	9	0.56	93	5.81	1
5	Fritz et al. (2008)	9	0.64	77	5.50	4
6	Sam (2001)	8	0.38	115	5.48	2
7	Pitts (2009)	8	0.62	62	4.77	1
8	Ying and Liese (1994)	7	0.25	47	1.68	0
9	Perrucci and Hu (1995)	7	0.26	63	2.33	1
10	Zhang and Goodson (2011)	7	0.64	53	4.82	0
11	Sawir et al. (2012)	7	0.70	50	5.00	5
12	Wang and Hannes (2014)	7	0.88	27	3.38	3
13	Akhtar and Kröner-Herwig (2015)	7	1.00	31	4.43	2
14	Yu and Shen (2012)	6	0.60	31	3.10	4
15	Yu (2013)	6	0.67	15	1.67	3
16	Young and Schartner (2014)	6	0.75	21	2.63	4
17	Rui and Wang (2015)	6	0.86	31	4.43	4
18	Zimmermann (1995)	5	0.19	62	2.30	2
19	Pedersen et al. (2011)	5	0.45	36	3.27	3
20	Hotta and Ting-Toomey (2013)	5	0.56	39	4.33	2

All indices retrieved from HistCite: *TLC*, total local citations received; *TLC/t*, average local citations received per year; *TGC*, total global citations received; *TGC/t*, average global citations received per year; *TLC/e*, trending local citations at the end of the period covered

**Table 3:** Future research questions

Research stream		Research questions	Author(s)
1. Second-language proficiency	1	How does second-language proficiency shape students’ adaptation in non-Anglophone countries (e.g., Russia, Japan, Spain)?	Fritz et al., (2008)
	2	What are the implications of students’ cultural backgrounds, education, and studied disciplines for the relationship between host-language proficiency and adaptation?	Sawir et al., (2012)
	3	What is the role of English as a lingua franca for students’ adaptation in non-Anglophone countries (e.g., China)? Is English proficiency sufficient for students’ adaptation and well-being? Is host-language proficiency necessary for adaptation processes?	*Authors*
2. Interactions in the host country	4	How are intercultural communication and cultural and identity transition processes related in the experience of long-term vs. short-term students?	Pitts (2009)
	5	Using longitudinal research, how is the development of friendships related to the adaptation process? How does the use of social media contribute to the adaptation and maintenance of friendships?	Hotta and Ting-Toomey (2013)
	6	How do interactions with hosts and co-nationals impact students’ cultural adaptation? Do interactions with co-nationals cause or result from a student’s adaptation difficulties?	Pedersen et al., (2011)
	7	What patterns of creating social networks facilitate/hamper adaptation? What role does the second language play in the creation of social networks? What is the impact of gender and students’ interactions with other international students on their adaptation and dealing with uncertainty and anxiety, and how does it differ from the impact of hosts and co-nationals?	Rui and Wang, (2015)
	8	What are the mediating/moderating effects of social interactions and social connectedness with co-nationals on students’ psychosocial adaptation?	Zhang and Goodson, (2011)
	9	What factors besides global competence (e.g., demographic factors, personality, social support, social acceptance, interpersonal relationships) contribute to the relationship between English proficiency and connectedness in an international community?	Meng et al. (2018)
	10	What is the role of agentic concepts (e.g., mindfulness, identity, flexibility) in shaping students’ willingness to communicate in the second language? How do difficulties related to academic forums (e.g., a lack of class cohesiveness) influence that willingness, intercultural communication in class, and academic and general adaptation?	Gallagher, (2013)
	11	What is the role of students’ pre-dispositional variables, motivations and interests (e.g., in developing language skills vs. other skills) in shaping the relationship between second-language proficiency and adaptation?	Young and Schartner ,(2014)
	12	Using experimental or dyadic interactional study designs, what is the role of host nationals’ stereotypical perceptions of international students in their willingness to communicate with these students?	Ruble and Zhang (2013)
	13	Using longitudinal research, how do integrative motivation and second-language competence interact to contribute to students’ academic and sociocultural adaptation?	Yu ,(2013)
	14	How are foreign-language proficiency, social connectedness, and socialization with hosts and co-nationals related to adaptation difficulties among students at different stages of postsecondary education?	Duru and Poyrazli ,(2011)
	15	How did the Covid-19 pandemic-induced online learning affect students’ intercultural interactions and cultural adaptation? How do online interactions with peers and teachers shape students’ adaptation? How does participation in online classes affect students’ confidence in using the second language, as well as their motivation and willingness to engage in intercultural communication in class? What is the role and scope of asynchronous communication in facilitating students’ intercultural interactions? What is the online learning experience of vulnerable students who lack comfortable study conditions, a reliable internet infrastructure, or attend classes from distant locations across different time zones?	*Authors*

The most trending article (TLCe = 7) was a quantitative study by Duru and Poyrazli (2011) who considered the role of social connectedness, perceived discrimination, and communication with locals and co-nationals in the sociocultural adaptation of Turkish students in the USA. The second article with the most trending local citations (TLCe = 5) was a qualitative study by Sawir et al. (2012) who focused on host-language proficiency as a barrier to sociocultural adaptation and communication in the experience of students in Anglophone countries.

### Keyword co-occurrence analysis

**Fig. 3:**
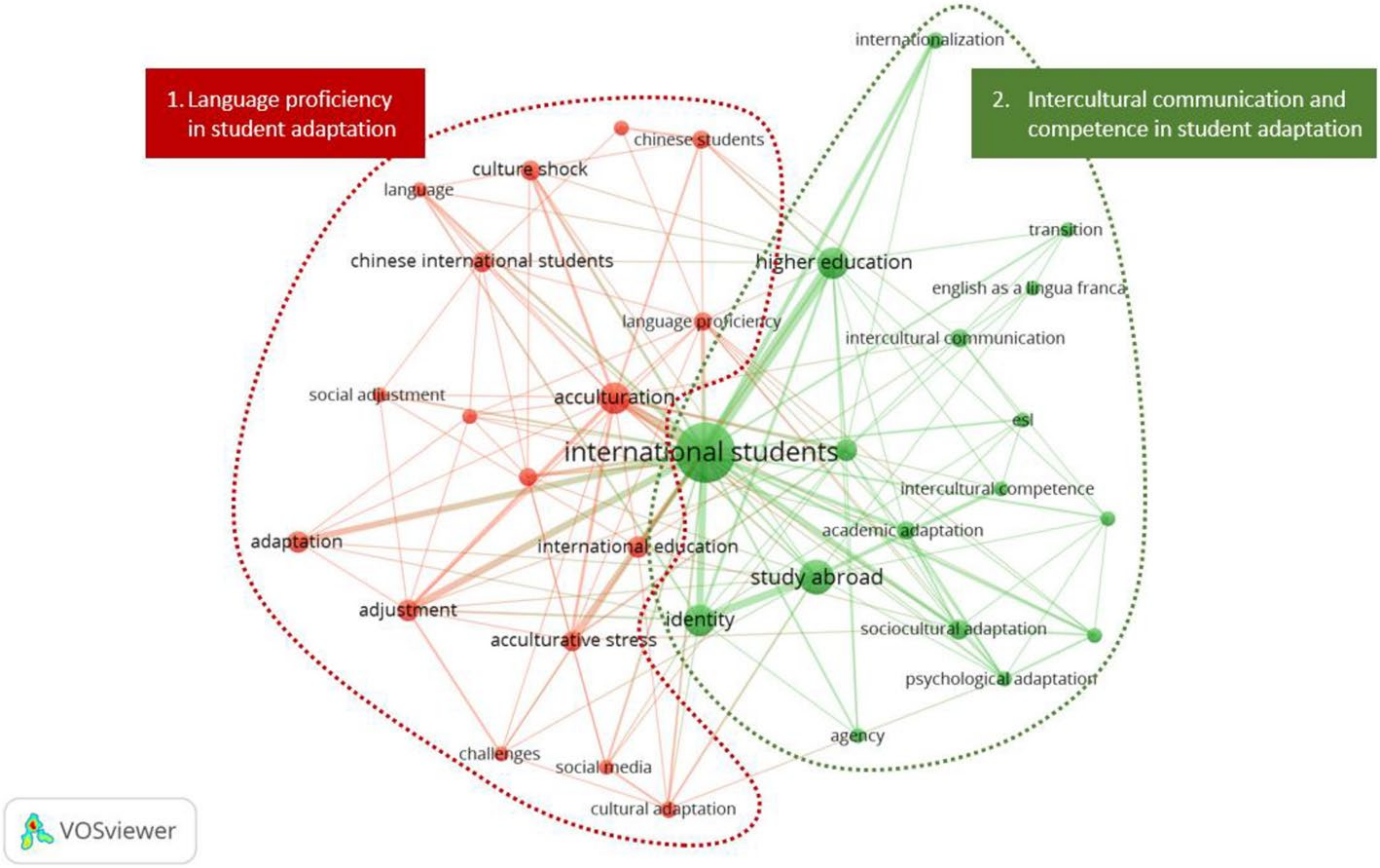
Authors’ keyword co-occurrence analysis ( Source: VOS)

We addressed research question 2 regarding the thematic structure of the research in the field by analyzing the authors’ keyword co-occurrences to establish the thematic structure of the field (Bretas and Alon, 2021; Donthu et al., 2020). Figure 3 depicts the network of keywords that occurred together in at least five articles between 1994 and 2021. The nodes represent keywords, the edges represent linkages among the keywords, and the proximity of the nodes and the thickness of the edges represent how frequently the keywords co-occurred (Donthu et al., 2020). The analysis yielded two even clusters with 17 keywords each. Cluster 1 represents the primary focus on the role of language proficiency in student adaptation. It includes keywords such as “language proficiency,” “adaptation,” “acculturative stress,” “culture shock,” and “challenges.” Cluster 2 represents the focus on the role of intercultural communication and competence in student adaptation. It includes keywords such as “intercultural communication,” “intercultural competence,” “academic/psychological/sociocultural adaptation,” and “transition.”

### Conceptual thematic map

**Fig. 4:**
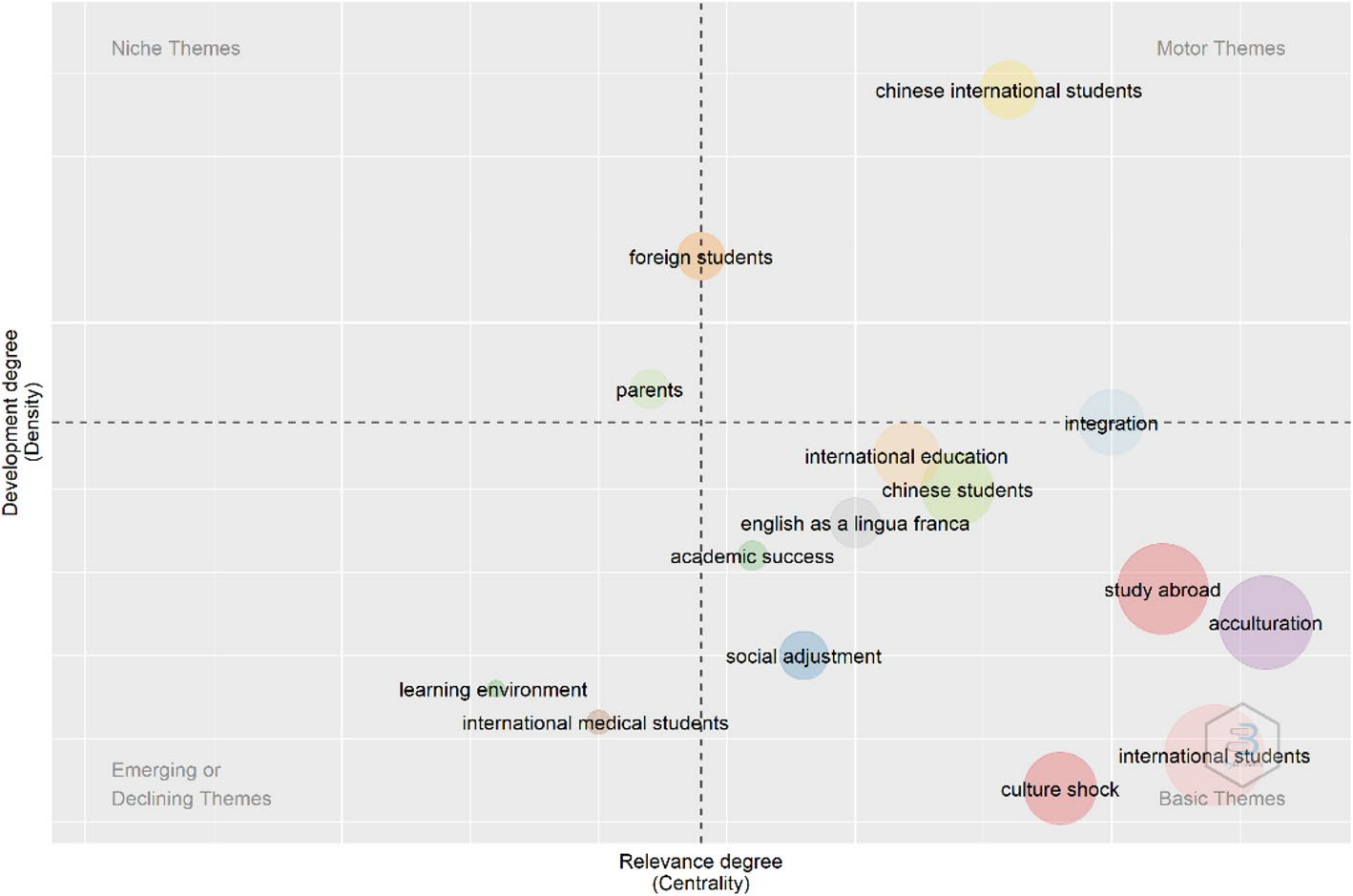
Conceptual thematic map ( Source: Biblioshiny)

Based on the authors’ keywords, we plotted a conceptual map (see Fig. 4) using two dimensions. The first is *density*, which indicates the degree of development of the themes as measured by the internal associations among the keywords. The second is *centrality*, which indicates the relevance of the themes as measured by the external associations among the keywords. The map shows four quadrants: (a) *motor themes* (high density and centrality), (b) *basic themes* (low density and high centrality), (c) *niche themes* (high density and low centrality), and (d) *emerging/declining themes* (low density and centrality) (Bretas & Alon, 2021). The analysis revealed that motor themes in the field are studies of Chinese students’ experiences and student integration. Unsurprisingly, the basic themes encompass most topics related to language in student adaptation. Research examining the perspective of the students’ parents with regard to their children’s overseas experience exemplifies a niche theme. Finally, “international medical students” and “learning environment” unfold as emerging/declining themes. To determine if the theme is emerging or declining, we analyzed bibliometric data on articles relating to medical students’ adaptation and students’ learning environment. We found that out of 19 articles on medical students published in 13 journals (10 medicine/public health-related), 15 (79%) articles were published over the last five years (2016–2021), which clearly suggests an emerging trend. The analysis of authors’ keywords yielded only three occurrences of the keyword “learning environment” in articles published in 2012, 2016, and 2020, which may suggest an emerging trend. To further validate this result, we searched for this keyword in titles and abstracts and identified eight relevant articles published between 2016 and 2020, which supports the emerging trend.

### Citation mapping: research streams

We addressed research question 3 regarding the leading research streams investigating language and communication in student adaptation by using co-citation mapping techniques to reveal how the articles in our dataset are co-cited over time. To produce meaningful results that would not trade depth for breadth in our large dataset (313 articles), we limited the search to articles with TGC ≥ 10 and TLC ≥ 3. These thresholds yielded the 31 articles (10% of the dataset) that are most frequently cited within and outside the dataset, indicating their driving force in the field. We analyzed these 31 articles further because their number corresponds with the suggested range of the most-cited core articles for mapping in HistCite (Garfield et al., 2006).

**Fig. 5:**
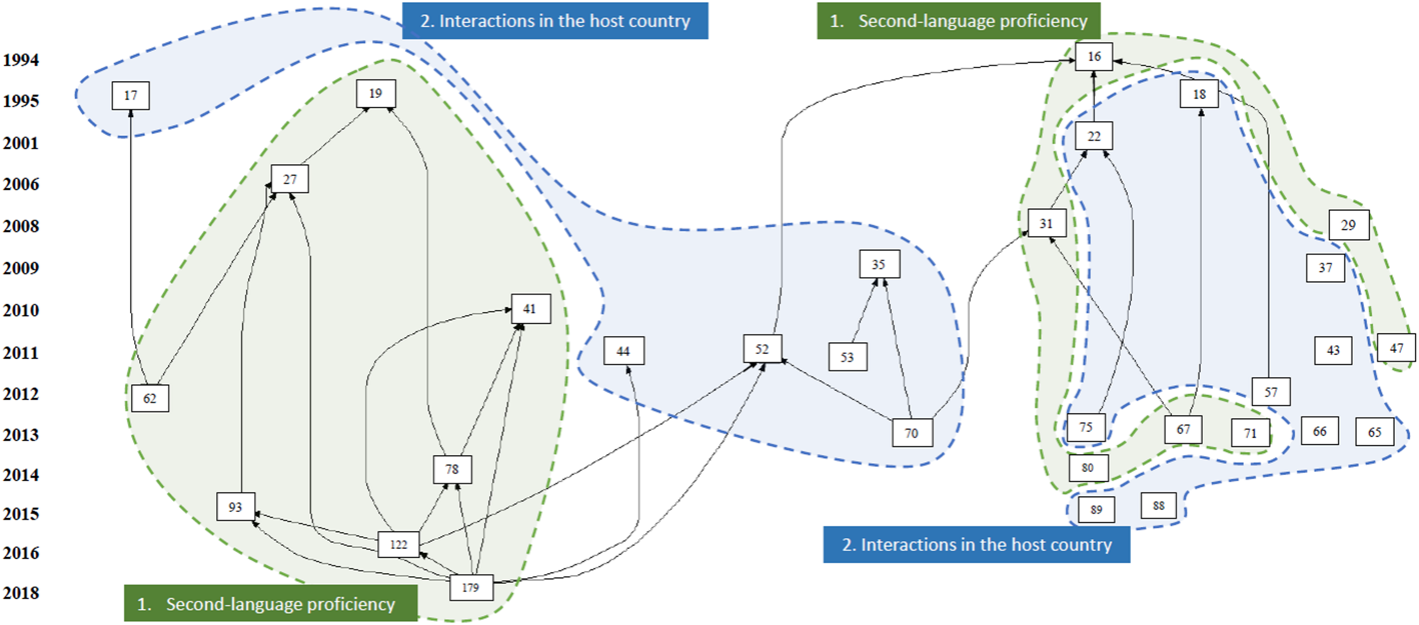
Citation mapping of articles on language and communication in student adaptation ( Source: HistCite)

Figure 5 presents the citation mapping of these 31 articles. The vertical axis shows how the articles have been co-cited over time. Each node represents an article, the number in the box represents the location of the article in the entire dataset, and the size of the box indicates the article’s impact in terms of TLCs. The arrows indicate the citing direction between two articles. A closer distance between two nodes/articles indicates their similarity. Ten isolated articles in Fig. 5 have not been co-cited by other articles in the subsample of 31 articles.

A content analysis of these 31 articles points to two major and quite even streams in the field: (a) “second-language proficiency” (16 articles) and (b) “interactions in the host country” involving second-language proficiency, communication competence, intercultural communication, and other factors (15 articles). We clustered the articles based on similar conceptualizations of language and communication and their role in student adaptation. As Fig. 5 illustrates, the articles formed distinct but interrelated clusters. The vertical axis indicates that while studies focusing solely on second-language proficiency and host-country interactions have developed relatively concurrently throughout the entire timespan, a particular interest in host-country interactions occurred in the second decade of research within the field (between 2009 and 2013). The ensuing sections present the results of the content analysis of the studies in each research stream, discussing the results in light of the major theories outlined before.

## Content analysis

**Fig. 6:**
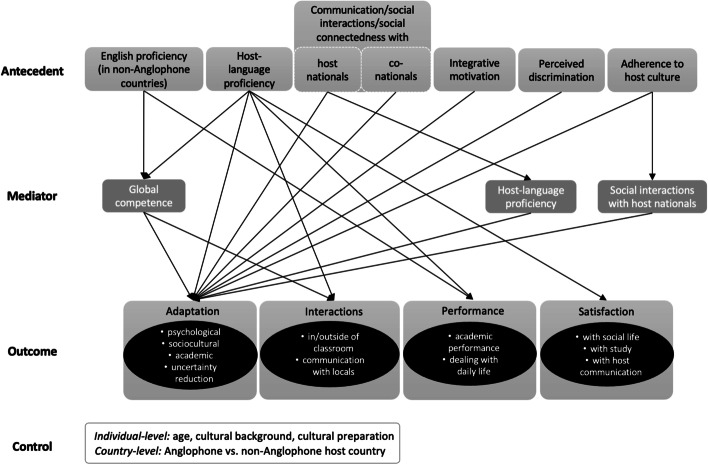
A concept map synthesizing research on language and communication in student adaptation

We sought to answer research question 4 regarding the effects of language and communication on student adaptation by synthesizing the literature within the previously established two research streams. The concept map in Fig. 6 illustrates the predictive effects of second-language proficiency and host-country interactions on various adaptation domains. Table 4 in the Appendix presents a detailed description of the synthesis and lists studies reporting these effects, underscoring inconclusive results.

### Second-language proficiency

This research stream focuses on language barriers and the role of foreign-language proficiency in student adaptation. Having host-language proficiency predicts less acculturative stress (Akhtar and Kröner-Herwig, 2015), while limited host-language proficiency inhibits communication with locals and academic integration (Cao et al., 2016). These results are in line with the acculturation theory (Berry, 1997, 2005; Ward et al., 2001) and the communication and cross-cultural adaptation theory (Kim, 2001). Cross (1995) suggested that social skills predict sociocultural rather than psychological (perceived stress, well-being) adaptation (Searle and Ward, 1990). Indeed, several qualitative studies have explained that the language barrier affects sociocultural adaptation by preventing students from establishing contacts with host nationals (Wang and Hannes, 2014), developing meaningful relationships (Sawir et al., 2012), and limiting occasions for cultural learning (Trentman, 2013), supporting the acculturation theory (Anderson, 1994; Church, 1982; Searle and Ward, 1990).

Moreover, insufficient host-language proficiency reduces students’ satisfaction by hampering their communication, socialization, and understanding of lectures in academic contexts (Campbell and Li, 2008). Similarly, language affects academic adaptation in students who have difficulty communicating with domestic students (Young and Schartner, 2014) or when used as a tool in power struggles, limiting students’ opportunities to speak up in class and participate in discussions or decision-making (Shi, 2011). Students who have limited host-language proficiency tend to interact with other international students, which exacerbates their separation from domestic students (Sawir et al., 2012). These findings again confirm the theories of acculturation (Berry, 1997; Ward et al., 2001) and communication and cross-cultural adaptation (Kim, 2001).

With regard to the acculturation theory (Berry, 1997; Ward and Kennedy, 1999), we found inconclusive results concerning the impact of foreign-language skills on students’ satisfaction and adaptation. Specifically, some studies (e.g., Sam, 2001; Ying and Liese, 1994) found this effect to be non-significant when tested in regression models. One explanation for this result might be the indirect effect of language on adaptation. For instance, Yang et al. (2006) established that host-language proficiency mediated the relationship between contact with host nationals and the psychological and sociocultural adjustment of students in Canada. Swami et al. (2010) reported that better host-language skills among Asian students in Britain predicted their adaptation partly because they had more contacts with host nationals. In turn, Meng et al. (2018) found that the relationship between foreign-language proficiency and social and academic adaptation was fully mediated by global competence (understood as “intercultural competence” or “global mindset”) in Chinese students in Belgium.

### Interactions in the host country

The second research stream comprises studies taking a broader look at language and communication in student adaptation by considering both individual and social interaction contexts: second-language (host-language and English) proficiency; willingness to communicate in the second language; communication interactions with domestic and international students, host nationals, and co-nationals; social connectedness (i.e., a subjective awareness of being in a close relationship with the social world; Lee and Robbins, 1998; and integrative motivation (i.e., a positive affective disposition towards the host community; Yu, 2013.

Host-language proficiency predicts academic (Hirai et al., 2015; Yu, 2013), psychological (Hirai et al., 2015; Rui and Wang, 2015), and sociocultural adaptation (Brown, 2009; Duru and Poyrazli, 2011), confirming the acculturation theory (Ward et al., 2001). However, although some studies (Hirai et al., 2015; Yu, 2013) confirmed the impact of host-language proficiency on academic adaptation, they found no such impact on sociocultural adaptation. Yu’s (2013) study reported that sociocultural adaptation depends on academic adaptation rather than on host-language proficiency. Moreover, host-language proficiency increases the students’ knowledge of the host culture, reduces their uncertainty, and promotes intercultural communication (Gallagher, 2013; Rui and Wang, 2015), supporting the central aspects of the AUM theory (Gudykunst, 2005).

In turn, by enabling communication with academics and peers, second-language proficiency promotes academic (Yu and Shen, 2012) and sociocultural adaptation, as well as social satisfaction (Perrucci and Hu, 1995). It also increases the students’ willingness to communicate in non-academic contexts. This willingness mediates the relationship between second-language proficiency and cross-cultural difficulties among Asian students in England (Gallagher, 2013). This finding may explain inconclusive results concerning the relationship between second-language proficiency and cultural adaptation. It appears that second-language proficiency alone is insufficient for successful adaptation. This proficiency should be coupled with the students’ willingness to initiate intercultural communication to cope with communication and cultural difficulties, which is compatible with both the AUM theory and Kim’s (2001) communication and cross-cultural adaptation theory.

As mentioned before, host-language proficiency facilitates adaptation through social interactions. Research demonstrates that communication with domestic students predicts academic satisfaction (Perrucci and Hu, 1995) and academic adaptation (Yu and Shen, 2012), confirming Kim’s (2001) theory. Moreover, the frequency of interaction (Zimmermann, 1995) and direct communication with host nationals (Rui and Wang, 2015) predict adaptation and reduce uncertainty, supporting the AUM theory. Zhang and Goodson (2011) found that social interactions with host nationals mediate the relationship between adherence to the host culture and sociocultural adaptation difficulties, confirming the acculturation theory (Berry, 1997), the intergroup contact theory (Allport, 1954; Pettigrew, 2008), and the culture learning approach in acculturation theory (Ward et al., 2001).

In line with the intergroup contact theory, social connectedness with host nationals predicts psychological and sociocultural adaptation (e.g., Hirai et al., 2015; Zhang and Goodson, 2011), confirming the sojourner adjustment framework (Church, 1982) and extending the acculturation framework (Ward and Kennedy, 1999) that recognizes the relevance of social connectedness for sociocultural adaptation only.

Research on interactions with co-nationals has produced inconclusive results. Some qualitative studies (Pitts, 2009) revealed that communication with co-nationals enhances students’ sociocultural adaptation and psychological and functional fitness for interacting with host nationals. Consistent with Kim’s (2001) theory, such communication may be a source of instrumental and emotional support for students when locals are not interested in contacts with them (Brown, 2009). Nonetheless, Pedersen et al. (2011) found that social interactions with co-nationals may cause psychological adjustment problems (e.g., homesickness), contradicting the acculturation theory (Ward and Kennedy, 1994), or increase their uncertainty (Rui and Wang, 2015), supporting the AUM theory.

### Avenues for future research

We addressed research question 5 regarding future research directions through a content analysis of the 31 most impactful articles in the field. Importantly, all 20 trending articles listed in Table 1 were contained in the set of 31 articles. This outcome confirms the relevance of the results of the content analysis. We used these results as the basis for formulating the research questions we believe should be addressed within each of the two research streams. These questions are listed in Table 3.

Research has focused primarily on the experience of Asian students in Anglophone countries (16 out of 31 most impactful articles), with Chinese students’ integration being the motor theme. This is not surprising given that Asian students account for 58% of all international students worldwide (OECD, 2021b). In addition, Anglophone countries have been the top host destinations for the last two decades. The USA, the UK, and Australia hosted 49% of international students in 2000, while the USA, the UK, Canada, and Australia hosted 47% of international students in 2020 (Project Atlas, 2020). This fact raises the question of the generalizability of the research results across cultural contexts, especially given the previously identified cultural variation in student adaptation (Fritz et al., 2008). Thus, it is important to study the experiences of students in underexplored non-Anglophone host destinations that are currently gaining in popularity, such as China, hosting 9% of international students worldwide in 2019, France, Japan, or Spain (Project Atlas, 2020). Furthermore, future research in various non-Anglophone countries could precisely define the role of English as a lingua franca vs. host-language proficiency in international students’ experience.

The inconsistent results concerning the effects of communication with co-nationals on student adaptation (e.g., Pedersen et al., 2011; Pitts, 2009) indicate that more contextualized research is needed to determine if such communication is a product of or a precursor to adaptation difficulties (Pedersen et al., 2011). Given the lack of confirmation of the acculturation theory (Ward and Kennedy, 1994) or the communication and cross-cultural adaptation theory (Kim, 2001) in this regard, future research could cross-check the formation of students’ social networks with their adaptation trajectories, potentially using other theories such as social network theory to explain the contradictory results of empirical research.

Zhang and Goodson (2011) showed that social connectedness and social interaction with host nationals predict both psychological and sociocultural adaptation. In contrast, the sojourner adjustment framework (Ward and Kennedy, 1999) considered their impact on sociocultural adaptation only. Thus, future research should conceptualize the interrelationships among social interactions in the host country and various adaptation domains (psychological, sociocultural, and academic) more precisely.

Some studies (Brown, 2009; Gallagher, 2013; Rui and Wang, 2015) confirm all of the major adaptation theories in that host-language proficiency increases cultural knowledge and the acquisition of social skills, reduces uncertainty and facilitates intercultural communication. Nevertheless, the impact of language on sociocultural adaptation appears to be a complex issue. Our content analysis indicated that sociocultural adaptation may be impacted by academic adaptation (Yu, 2013) or does not occur when students do not engage in meaningful interactions with host nationals (Ortaçtepe, 2013). To better capture the positive sociocultural adaptation outcomes, researchers should take into account students’ communication motivations, together with other types of adaptation that may determine sociocultural adaptation.

Next, in view of some research suggesting the mediating role of second-language proficiency (Yang et al., 2006), contacts with host nationals (Swami et al., 2010), and students’ global competence (Meng et al., 2018) in their adaptation, future research should consider other non-language-related factors such as demographic, sociocultural, and personality characteristics in student adaptation models.

Finally, the conceptual map of the field established the experiences of medical students and the learning environment as an emerging research agenda. We expect that future research will focus on the experience of other types of students such as management or tourism students who combine studies with gaining professional experience in their fields. In terms of the learning environment and given the development and growing importance of online learning as a result of the Covid-19 pandemic, future research should explore the effects of remote communication, both synchronous and asynchronous, in online learning on students’ adaptation and well-being.

## Conclusion

This article offers an objective approach to reviewing the current state of the literature on language and communication in student adaptation by conducting a bibliometric analysis of 313 articles and a content analysis of 31 articles identified as the driving force in the field. Only articles in English were included due to the authors’ inability to read the identified articles in Russian, Spanish, or Chinese. Future research could extend the data search to other languages.

This review found support for the effects of language of communication on student adaptation, confirming major adaptation theories. Nevertheless, it also identified inconsistent results concerning communication with co-nationals and the complex effects of communication with host nationals. Thus, we suggested that future research better captures the adaptation outcomes by conducting contextualized research in various cultural contexts, tracking the formation of students’ social networks, and precisely conceptualizing interrelations among social interactions in the host country and different adaptation domains. Researchers should also consider students’ communication motivations and the mediating role of non-language-related factors in student adaptation models.

## Appendix

**Table 4:** A synthesis of the literature on language and communication in student adaptation

Effects of second-language proficiency and interactions in the host country	
***Antecedent***	***Outcome***
English proficiency (in non-Anglophone countries)	( +) Understanding academic English/following lectures (Wang and Hannes, 2014) ( +) Dealing with daily life tasks (Cao et al., 2016)
Host-language proficiency	**Adaptation and integration domain** ( +) Adaptation/adjustment • sociocultural (Brown, 2009; Duru and Poyrazli, 2011; Hirai et al., 2015; Khawaja & Stallman, 2011; Rui and Wang, 2015; Sawir et al., 2012; Swami et al., 2010; Wang and Hannes, 2014; Yang et al., 2006; Yu and Shen, 2012; Zimmerman, 1995); nonsignificant effect in a model with academic adaptation (Yu, 2013) • psychological (Rui and Wang, 2015; Yang et al., 2006); nonsignificant effect in Hirai et al. (2015) • academic (Khawaja & Stallman, 2011; Shi, 2011; Yu, 2013; Yu and Shen, 2012); nonsignificant effect when tested in a model (Sam, 2001; Ying and Liese, 1994) ( +) Academic integration (Cao et al., 2016) ( +) Uncertainty reduction (Gallagher, 2013; Rui and Wang, 2015) ( +) Coping with cross-cultural stressors (Gallagher, 2013) (-) Acculturative stress (Akhtar and Kröner-Herwig, 2015) (-) ‘Marginalization’ acculturation style (Pedersen et al., 2011) **Satisfaction domain** ( +) Satisfaction with • social life (Perrucci and Hu, 1995) • study (Campbell and Li, 2008; Perrucci and Hu, 1995) • communication in the host culture (Zimmerman, 1995) **Performance domain** ( +) Academic performance (Young and Schartner, 2014) ( +) Understanding lectures (Campbell and Li, 2008) ( +) Dealing with daily life tasks (Cao et al., 2016) **Interactions domain** ( +) Communication with • locals (Brown, 2009; Campbell and Li, 2008; Sawir et al., 2012) • domestic students (Campbell and Li, 2008; Perrucci and Hu, 1995; Trentman, 2013) • teachers (Campbell and Li, 2008) ( +) Willingness to communicate outside the classroom (Gallagher, 2013) ( +) Classroom interactions (Young and Schartner, 2014)
Communication/social interactions with host nationals	( +) Sociocultural adaptation (Duru and Poyrazli, 2011; Khawaja & Stallman, 2011; Zhang and Goodson, 2011) ( +) Academic adaptation (Khawaja & Stallman, 2011; Yu and Shen, 2012) & academic achievement (Khawaja & Stallman, 2011) ( +) Cultural adaptation (Zimmerman, 1995) ( +) Uncertainty reduction (Rui and Wang, 2015) ( +) ‘Integration’ & ‘assimilation’ acculturation style (Pedersen et al., 2011) (-) ‘Marginalization’ acculturation style (Pedersen et al., 2011)
Social connectedness with host nationals	( +) Sociocultural adjustment (Duru and Poyrazli, 2011; Hirai et al., 2015; Zhang and Goodson, 2011) ( +) Psychological adjustment (Hirai et al., 2015; Zhang and Goodson, 2011)
Integrative motivation	( +) Sociocultural & academic adaptation (Yu and Shen, 2012)
Adherence to host culture	( +) Sociocultural & psychological adjustment (Zhang and Goodson, 2011)
Perceived discrimination	( +) Adaptation difficulties (Duru and Poyrazli, 2011)
Communication/social interactions with co-nationals	(+) Sociocultural adaptation, psychological and functional fitness to interacting with host nationals (Pitts, 2009) ( +) Negative psychological adjustment (homesickness/feeling out of place) (Pedersen et al., 2011); however, weak in-group identification with co-nationals may amplify loneliness (Ortaçtepe, 2013) ( +) ‘Separation’ acculturation style (Pedersen et al., 2011) (-) ‘Assimilation’ & ‘marginalization’ acculturation style (Pedersen et al., 2011) (-) Uncertainty reduction (Rui and Wang, 2015)
Social connectedness with co-nationals	( +) Psychological adjustment (Hirai et al., 2015)
**Controls (antecedents of stress, language, and adaptation difficulty)**	
**At individual level:** •Age (Akhtar and Kröner-Herwig, 2015) •Cultural background: Asian vs. European students (Fritz et al., 2008); Interdependent vs. independent self-construal (Cross, 1995) •Cultural preparation: intercultural communication training (Young and Schartner, 2014) **At country level:** • Anglophone vs. non-Anglophone host country (Cao et al., 2016)	
**Mediators**	
•Host-language & English proficiency (in a non-Anglophone country) **global competence** social & academic adaptation, social connectedness with international students (Meng et al., 2018) •Contact with host nationals **host-language proficiency** psychological & sociocultural adjustment (Yang et al., 2006) •Adherence to the host culture **social interactions with host nationals** sociocultural adjustment (Zhang & Goodson, 2011)	

(+) and (-) signify the positive and negative direction of the effect, respectively.

## References

[CR1] Akhtar M, Kröner-Herwig B (2015). Acculturative stress among international students in context of socio-demographic variables and coping styles. Current Psychology.

[CR2] Allport, G. W. (1954). *The nature of prejudice*. Perseus Books.

[CR3] Alon I, Anderson J, Munim ZH, Ho A (2018). A review of the internationalization of Chinese enterprises. Asia Pacific Journal of Management.

[CR4] Anderson LE (1994). A new look at an old construct: Cross-cultural adaptation. International Journal of Intercultural Relations.

[CR5] Andrade MS (2006). International students in English-speaking universities. Journal of Research in International Education.

[CR6] Benzie HJ (2010). Graduating as a “native speaker”: International students and English language proficiency in higher education. Higher Education Research and Development.

[CR7] Berry JW (1997). Immigration, acculturation, and adaptation. Applied Psychology.

[CR8] Berry JW (2005). Acculturation: Living successfully in two cultures. International Journal of Intercultural Relations.

[CR9] Black JS (1990). The relationship of personal characteristics with the adjustment of Japanese expatriate managers. Management International Review.

[CR10] Bretas VPG, Alon I (2021). Franchising research on emerging markets: Bibliometric and content analyses. Journal of Business Research.

[CR11] Brown L (2009). Using an ethnographic approach to understand the adjustment journey of international students at a university in England. Advances in Culture, Tourism and Hospitality Research.

[CR12] Burr V (2006). An introduction to social constructionism.

[CR13] Campbell J, Li M (2008). Asian students’ voices: An empirical study of Asian students’ learning experiences at a New Zealand University. Journal of Studies in International Education.

[CR14] Cao C, Zhu C, Meng Q (2016). An exploratory study of inter-relationships of acculturative stressors among Chinese students from six European union (EU) countries. International Journal of Intercultural Relations.

[CR15] Church AT (1982). Sojourner adjustment. Psychological Bulletin.

[CR16] Cross SE (1995). Self-construals, coping, and stress in cross-cultural adaptation. Journal of Cross-Cultural Psychology.

[CR17] Donthu N, Kumar S, Ranaweera C, Sigala M, Sureka R (2020). Journal of Service Theory and Practice at age 30: Past, present and future contributions to service research. Journal of Service Theory and Practice.

[CR18] Duru E, Poyrazli S (2011). Perceived discrimination, social connectedness, and other predictors of adjustment difficulties among Turkish international students. International Journal of Psychology.

[CR19] Fritz MV, Chin D, DeMarinis V (2008). Stressors, anxiety, acculturation and adjustment among international and North American students. International Journal of Intercultural Relations.

[CR20] Gallagher HC (2013). Willingness to communicate and cross-cultural adaptation: L2 communication and acculturative stress as transaction. Applied Linguistics.

[CR21] Garfield E, Paris SW, Stock WG (2006). HistCite™ : A software tool for informetric analysis of citation linkage. Information.

[CR22] Gudykunst, W. B., & Hammer, M. R. (1988). Strangers and hosts—An uncertainty reduction based theory of intercultural adaptation. In Y. Y. Kim & W. B. Gudykunst (Eds.), *Cross-cultural adaptation: Current approaches* (Vol. 11, pp. 106–139). Sage.

[CR23] Gudykunst WB, Gudykunst WB (2005). An anxiety/uncertainty management (AUM) theory of strangers’ intercultural adjustment. Theorizing about intercultural communication.

[CR24] Hirai R, Frazier P, Syed M (2015). Psychological and sociocultural adjustment of first-year international students: Trajectories and predictors. Journal of Counseling Psychology.

[CR25] Hope J (2020). Be aware of how COVID-19 could impact international students. The Successful Registrar.

[CR26] Hotta, J., & Ting-Toomey, S. (2013). Intercultural adjustment and friendship dialectics in international students: A qualitative study. *International Journal of Intercultural Relations, 37*(5), 550–566. 10.1016/j.ijintrel.2013.06.007

[CR27] Jackson J (2015). Becoming interculturally competent: Theory to practice in international education. International Journal of Intercultural Relations.

[CR28] Jing X, Ghosh R, Sun Z, Liu Q (2020). Mapping global research related to international students: A scientometric review. Higher Education.

[CR29] Khawaja, N. G., & Stallman, H. M. (2011). Understanding the coping strategies of international students: A qualitative approach. *Journal of Psychologists and Counsellors in Schools, 21*(2), 203–224. 10.1375/ajgc.21.2.203

[CR30] Kim, Y. Y. (2001). *Becoming intercultural: An integrative theory of communication and cross-cultural adaptation*. Sage.

[CR31] Kramsch C (1998). Language and culture.

[CR32] Lee RM, Robbins SB (1998). The relationship between social connectedness and anxiety, self-esteem, and social identity. Journal of Counseling Psychology.

[CR33] Marginson S (2014). Student self-formation in international education. Student Self-Formation in International Education.

[CR34] Meng Q, Zhu C, Cao C (2018). Chinese international students’ social connectedness, social and academic adaptation: The mediating role of global competence. Higher Education.

[CR35] Neuendorf, K. A. (2002). *The content analysis guidebook* (2nd ed.). Sage.

[CR36] OECD (2021a). *Education at a Glance 2021: OECD indicators*. Paris: OECD Publishing. 10.1787/b35a14e5-en.

[CR37] OECD (2021b). *What is the profile of internationally mobile students?*10.1787/5A49E448-EN

[CR38] Ortaçtepe D (2013). “This is called free-falling theory not culture shock!”: A narrative inquiry on second language socialization. Journal of Language, Identity and Education.

[CR39] Pedersen ER, Neighbors C, Larimer ME, Lee CM (2011). Measuring sojourner adjustment among American students studying abroad. International Journal of Intercultural Relations.

[CR40] Perrucci R, Hu H (1995). Satisfaction with social and educational experiences among international graduate students. Research in Higher Education.

[CR41] Pettigrew TF (2008). Future directions for intergroup contact theory and research. International Journal of Intercultural Relations.

[CR42] Piller I (2007). Linguistics and Intercultural Communication. Language and Linguistics Compass.

[CR43] Pitts MJ (2009). Identity and the role of expectations, stress, and talk in short-term student sojourner adjustment: An application of the integrative theory of communication and cross-cultural adaptation. International Journal of Intercultural Relations.

[CR44] *Project Atlas*. (2020). https://iie.widen.net/s/rfw2c7rrbd/project-atlas-infographics-2020. Accessed 15 September 2021.

[CR45] Ruble, R. A., & Zhang, Y. B. (2013). Stereotypes of Chinese international students held by Americans. *International Journal of Intercultural Relations, 37*(2), 202–211. 10.1016/j.ijintrel.2012.12.004

[CR46] Rui JR, Wang H (2015). Social network sites and international students’ cross-cultural adaptation. Computers in Human Behavior.

[CR47] Sam DL (2001). Satisfaction with life among international students: An exploratory study. Social Indicators Research.

[CR48] Sawir E, Marginson S, Forbes-Mewett H, Nyland C, Ramia G (2012). International student security and English language proficiency. Journal of Studies in International Education.

[CR49] Searle W, Ward C (1990). The prediction of psychological and sociocultural adjustment during cross-cultural transitions. International Journal of Intercultural Relations.

[CR50] Shi X (2011). Negotiating power and access to second language resources: A study on short-term Chinese MBA students in America. Modern Language Journal.

[CR51] Smith RA, Khawaja NG (2011). A review of the acculturation experiences of international students. International Journal of Intercultural Relations.

[CR52] Swami V, Arteche A, Chamorro-Premuzic T, Furnham A (2010). Sociocultural adjustment among sojourning Malaysian students in Britain: A replication and path analytic extension. Social Psychiatry and Psychiatric Epidemiology.

[CR53] Trentman E (2013). Imagined communities and language learning during study abroad: Arabic Learners in Egypt. Foreign Language Annals.

[CR54] *UIS Statistics*. (2021). http://data.uis.unesco.org/Index.aspx?queryid=172#. Accessed 10 December 2021.

[CR55] Volet, S., & Jones, C. (2012). Cultural transitions in higher education: Individual adaptation, transformation and engagement. *Advances in Motivation and Achievement, 17*, 241–284. 10.1108/S0749-7423(2012)0000017012

[CR56] Wang Q, Hannes K (2014). Academic and socio-cultural adjustment among Asian international students in the Flemish community of Belgium: A photovoice project. International Journal of Intercultural Relations.

[CR57] Ward, C., Bochner, S., & Furnham, A. (2001). *The psychology of culture shock* (2nd ed.). Routledge.

[CR58] Ward C, Kennedy A (1993). Where’s the “culture” in cross-cultural transition? Comparative studies of sojourner adjustment. Journal of Cross-Cultural Psychology.

[CR59] Ward C, Kennedy A (1994). Acculturation strategies, psychological adjustment, and sociocultural competence during cross-cultural transitions. International Journal of Intercultural Relations.

[CR60] Ward C, Kennedy A (1999). The measurement of sociocultural adaptation. International Journal of Intercultural Relations.

[CR61] Yang RPJ, Noels KA, Saumure KD (2006). Multiple routes to cross-cultural adaptation for international students: Mapping the paths between self-construals, English language confidence, and adjustment. International Journal of Intercultural Relations.

[CR62] Ying Y, Liese LH (1994). Initial adjustment of Taiwanese students to the United States: The impact of postarrival variables. Journal of Cross-Cultural Psychology.

[CR63] Young TJ, Schartner A (2014). The effects of cross-cultural communication education on international students’ adjustment and adaptation. Journal of Multilingual and Multicultural Development.

[CR64] Yu B (2013). Asian international students at an Australian university: Mapping the paths between integrative motivation, competence in L2 communication, cross-cultural adaptation and persistence with structural equation modelling. Journal of Multilingual and Multicultural Development.

[CR65] Yu B, Shen H (2012). Predicting roles of linguistic confidence, integrative motivation and second language proficiency on cross-cultural adaptation. International Journal of Intercultural Relations.

[CR66] Zhang J, Goodson P (2011). Acculturation and psychosocial adjustment of Chinese international students: Examining mediation and moderation effects. International Journal of Intercultural Relations.

[CR67] Zimmermann S (1995). Perceptions of intercultural communication competence and international student adaptation to an American campus. Communication Education.

[CR68] Zupic I, Čater T (2015). Bibliometric methods in management and organization. Organizational Research Methods.

